# Joint angle estimation during shoulder abduction exercise using contactless technology

**DOI:** 10.1186/s12938-024-01203-5

**Published:** 2024-01-28

**Authors:** Ali Barzegar Khanghah, Geoff Fernie, Atena Roshan Fekr

**Affiliations:** 1 KITE Research Institute, Toronto Rehabilitation Institute, University Health Network, 550 University Ave, Toronto, M5G 2A2 ON Canada; 2 Institute of Biomedical Engineering, University of Toronto, 164 College St., Toronto, M5S 3G9 ON Canada; 3 Department of Surgery, University of Toronto, 149 College St., Toronto, M5T 1P5 ON Canada

**Keywords:** Tele-rehabilitation, Marker-less joint angle estimation, System validation, Motion capture, Calibration

## Abstract

**Background:**

Tele-rehabilitation, also known as tele-rehab, uses communication technologies to provide rehabilitation services from a distance. The COVID-19 pandemic has highlighted the importance of tele-rehab, where the in-person visits declined and the demand for remote healthcare rises. Tele-rehab offers enhanced accessibility, convenience, cost-effectiveness, flexibility, care quality, continuity, and communication. However, the current systems are often not able to perform a comprehensive movement analysis. To address this, we propose and validate a novel approach using depth technology and skeleton tracking algorithms.

**Methods:**

Our data involved 14 participants (8 females, 6 males) performing shoulder abduction exercises. We collected depth videos from an LiDAR camera and motion data from a Motion Capture (Mocap) system as our ground truth. The data were collected at distances of 2 m, 2.5 m, and 3.5 m from the LiDAR sensor for both arms. Our innovative approach integrates LiDAR with the Cubemos and Mediapipe skeleton tracking frameworks, enabling the assessment of 3D joint angles. We validated the system by comparing the estimated joint angles versus Mocap outputs. Personalized calibration was applied using various regression models to enhance the accuracy of the joint angle calculations.

**Results:**

The Cubemos skeleton tracking system outperformed Mediapipe in joint angle estimation with higher accuracy and fewer errors. The proposed system showed a strong correlation with Mocap results, although some deviations were present due to noise. Precision decreased as the distance from the camera increased. Calibration significantly improved performance. Linear regression models consistently outperformed nonlinear models, especially at shorter distances.

**Conclusion:**

This study showcases the potential of a marker-less system, to proficiently track body joints and upper-limb angles. Signals from the proposed system and the Mocap system exhibited robust correlation, with Mean Absolute Errors (MAEs) consistently below $10^\circ$. LiDAR’s depth feature enabled accurate computation of in-depth angles beyond the reach of traditional RGB cameras. Altogether, this emphasizes the depth-based system’s potential for precise joint tracking and angle calculation in tele-rehab applications.

## Introduction

### Background

Tele-rehabilitation (tele-rehab), the use of telecommunication technologies to deliver rehabilitation services remotely, has emerged as a promising solution to address the challenges posed by traditional rehabilitation services [1]. Despite still being in its infancy, the use of tele-rehab systems is rapidly increasing [2]. The important role of tele-rehab has been highlighted during the COVID-19 pandemic, when in-person visits have been restricted and demands for accessible and remote healthcare services have been raised [3]. The results of a survey conducted in Ontario in May 2020 showed that despite suggestions for a gradual return to in-person care, there was a significant desire to continue using virtual services even after in-person visits were resumed. This suggests that virtual care will have a significant impact on the healthcare system [4].

There are several potential benefits of tele-rehab over traditional, in-person rehabilitation programs:

1. Accessibility: Tele-rehab provides a secure alternative for individuals to receive rehabilitation services from the comfort of their homes. It has the potential to increase the accessibility of care services for underserved populations [1, 5]. Tele-rehab can make rehabilitation services more accessible to individuals living in rural, underdeveloped, or remote areas or those with mobility issues who may have difficulty traveling to a clinic.
2. Convenience: Tele-rehab allows individuals to receive treatment, reducing the need for time-off work or arranging transportation.
3. Cost-effectiveness: Tele-rehab can be more cost-effective than traditional rehabilitation, as it eliminates the need for travel and may require fewer resources to deliver the same level of care [6]. In addition, studies have shown that tele-rehab does not impose any extra costs compared to traditional care. For example, Nelson et al. [7] conducted a trial-based economic evaluation of tele-rehab versus traditional care after total hip replacement. This study showed that the average cost difference between tele-rehab and traditional care was not statistically significant; however, a slight difference existed, with tele-rehab being the cheaper alternative. They claimed that the average cost per person decreases by $28.90 when using tele-rehab compared to conventional therapy. Nevertheless, Hwang et al. [8] concluded that tele-rehab had a significantly lower cost per patient ($− 1590, CI: − 2822, − 359) than traditional care. Tele-rehab can also reduce the spread of infectious diseases by limiting the number of in-person visits to a healthcare facility. It provides a way for infected or at-risk patients to receive therapy while in isolation [9]. This reduction in infectious disease transmission also prevents some potential costs of the healthcare system.
4. Flexibility: Tele-rehab can be more flexible than traditional rehabilitation, as appointments can be scheduled at the convenience of the patient and the therapist [10].
5. Quality of care: Some studies have suggested that tele-rehab can be as effective as traditional rehabilitation in terms of patient care outcomes [10–13]. Furthermore, tele-rehab is capable of providing routine monitoring of patient activity [13], which adds a useful source of information for health professionals to gain a better understanding of the patient’s condition and progress.
6. Continuity of care: Tele-rehab can help improve continuity of care by allowing individuals to continue receiving treatment even if they are unable to physically attend appointments due to illness or other circumstances [14].
7. Enhanced communication: Tele-rehab can facilitate communication between patients, caregivers, and multiple therapists, allowing for a more holistic and coordinated approach to care [15].

Tele-rehab services include a variety of offerings, such as assessment, monitoring, prevention, intervention, supervision, education, consultation, and coaching [16].

### Identified gaps

While there have been advancements in tele-rehab technology, there are still challenges in the practical delivery of these services to patients. Many current tele-rehab platforms rely on videoconferencing or web-based communication, which require clinician supervision [1]. However, these platforms do not have the capability to analyze the patient’s movement patterns to provide guidance on exercise programs [17].

A recent systematic review of existing Motion Capture (Mocap) systems published between 2015 and 2020 reported that approximately half of these systems used a Kinect sensor (48%), while the rest used Inertial Measurement Units (IMUs), Microsoft HoloLens and other types of optical systems, as shown in [18].

Although Kinect is a useful contactless motion sensing device, its outputs may be subject to occlusion or poor performance under various light conditions [19]. In addition, the accuracy of the algorithms is limited to the resolution and the distance of the camera from the subject. Wearable technology, in the form of IMUs, is the second most popular approach. Previous studies in this field reported some limitations, such as the use of a large number of sensors (e.g., eight IMUs), which can affect the usability and cost of the system. Another challenge of using IMUs is that their precision degrades over time due to biases, drifts, and random noise, and therefore, they require frequent calibration to maintain their accuracy [20, 21]. Furthermore, studies have shown that most older adults are not compliant with using this technology and do not want to wear the devices [22].

### Focus and intent

Due to the existing constraints of current tele-rehabilitation technologies such as Kinect sensors and IMUs, there is a need for a novel platform that harnesses modern technologies. In this paper, we present a comparative analysis of 3D joint angle estimation in tele-rehabilitation, specifically focusing on the integration of a Light Detection and Ranging (LiDAR) camera [23] and two skeleton tracking Software Development Kits (SDKs), Mediapipe, and Cubemos. Similar to our peers in this field who have already validated their systems against a gold standard [24–27], we evaluate the performance of our proposed system against the Cortex Mocap system as the gold standard [28]. The Mocap system uses marker-based tracking techniques and provides high-accuracy measurements [29, 30]. Mocap is an expensive system that is typically used in restricted areas, such as laboratories [30, 31].

Our objective is to propose an automated tele-rehab platform by integrating LiDAR technology and advanced skeleton tracking SDKs, e.g., Mediapipe and Cubemos. We aim to validate the accuracy of our system in estimating three-dimensional joint angles by conducting a comprehensive comparison, using Mocap as the benchmark for ground truth. LiDAR sensors use laser-based depth perception to capture detailed 3D information of the human body. Leveraging LiDAR’s depth perception capabilities, our goal is to offer a noninvasive, marker-less, privacy-preserving solution for joint angle assessment, overcoming limitations associated with traditional motion capture systems.

## Results

### Comparison results

**Fig. 1:**
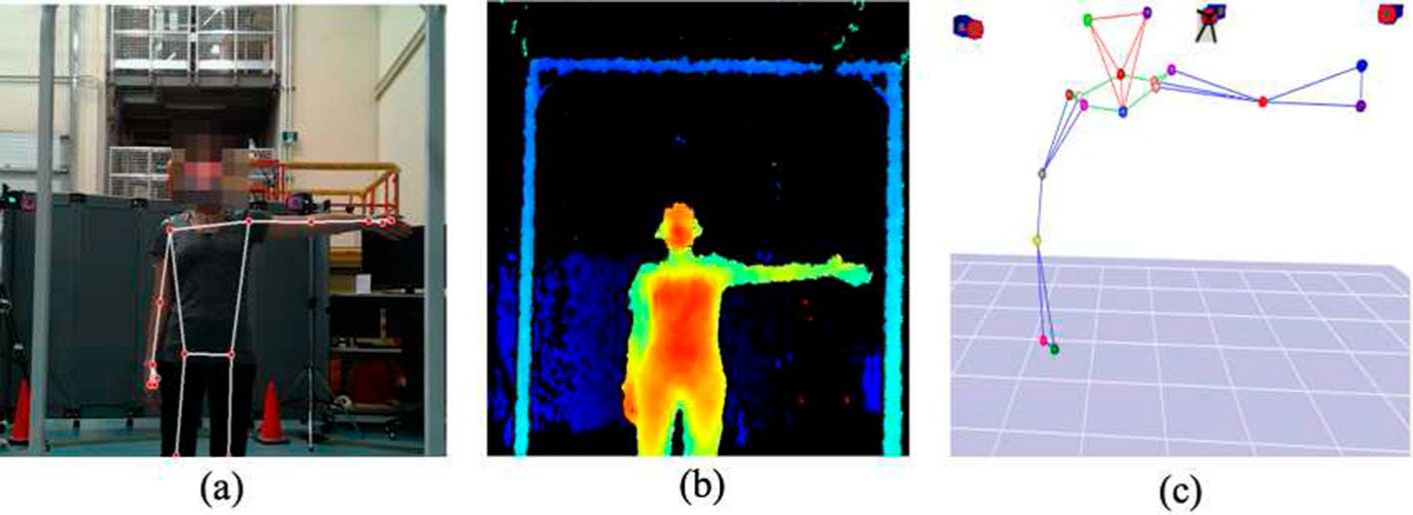
Sample frame: **a** RGB with Mediapipe skeleton tracking, **b** depth, and **c** Mocap at 2 m distance

Figure 1 shows samples of collected RGB, depth, and Mocap frames of the same time instance for S9 at a 2 m distance from the camera.

**Fig. 2:**
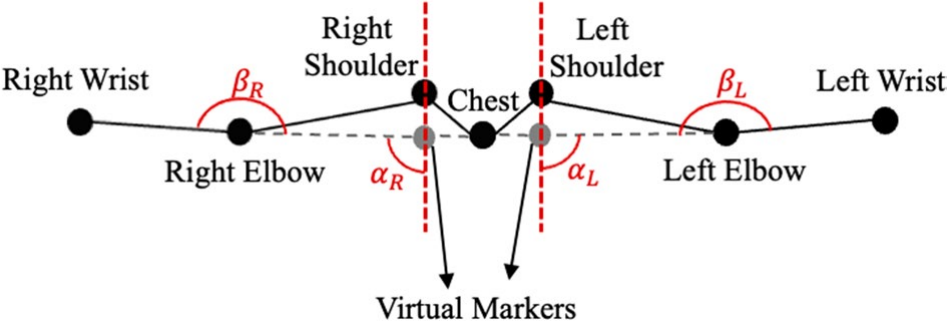
An example Mocap frame: virtual joints, shoulder ($\alpha$) and elbows ($\beta$) angles of S9 at full SAL, frontal view

**Fig. 3:**
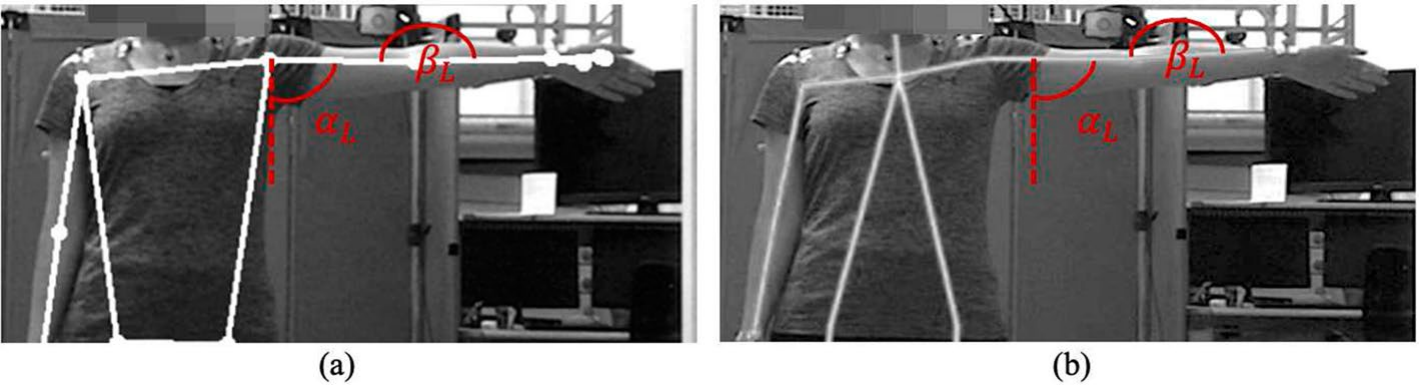
The (**a**) Mediapipe and (**b**) Cubemos frames showing $\alpha$ and $\beta$ angles of S9 at a full SAL

Before calculating the angles and conducting the comparisons, as shown in Figs. 2 and 3, we needed to create a virtual joint for each shoulder in Mocap to ensure that both systems measured the same joint angles. These new virtual markers are in the same longitude as the physical Mocap markers but have the same depth and height as the chest marker.

**Fig. 4:**
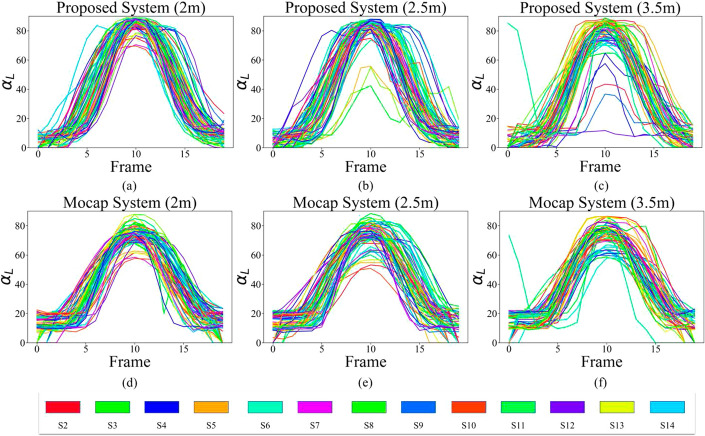
Signal $\alpha _{L}$ from proposed system at **a** 2 m, **b** 2.5 m, and **c** 3.5 m distances, and from Mocap at **d** 2 m, **e** 2.5 m, and **f** 3.5 m distances

**Fig. 5:**
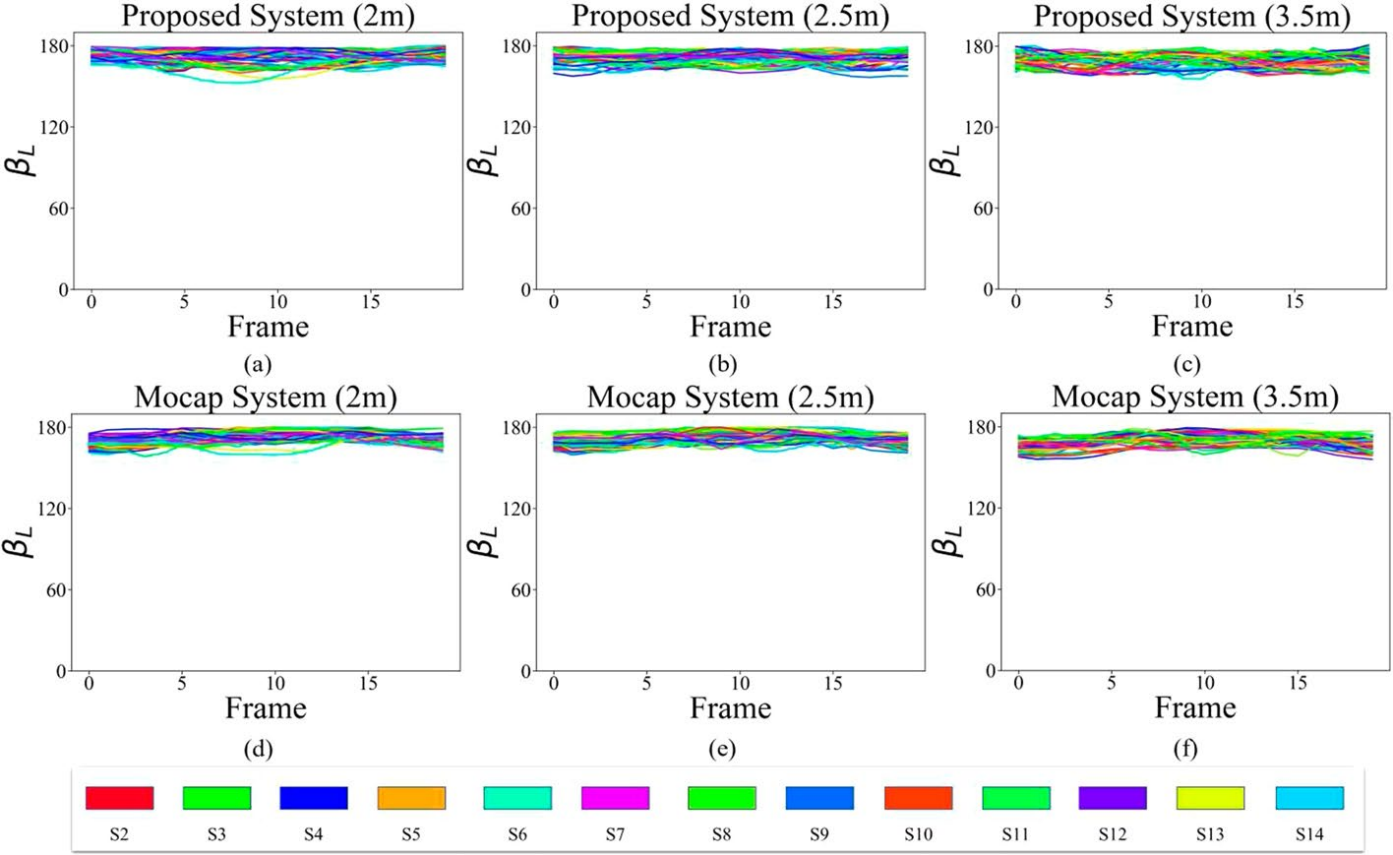
Signal $\beta _{L}$ from proposed system at **a** 2 m, **b** 2.5 m, and **c** 3.5 m distances, and from Mocap at **d** 2 m, **e** 2.5 m, and **f** 3.5 m distances

**Fig. 6:**
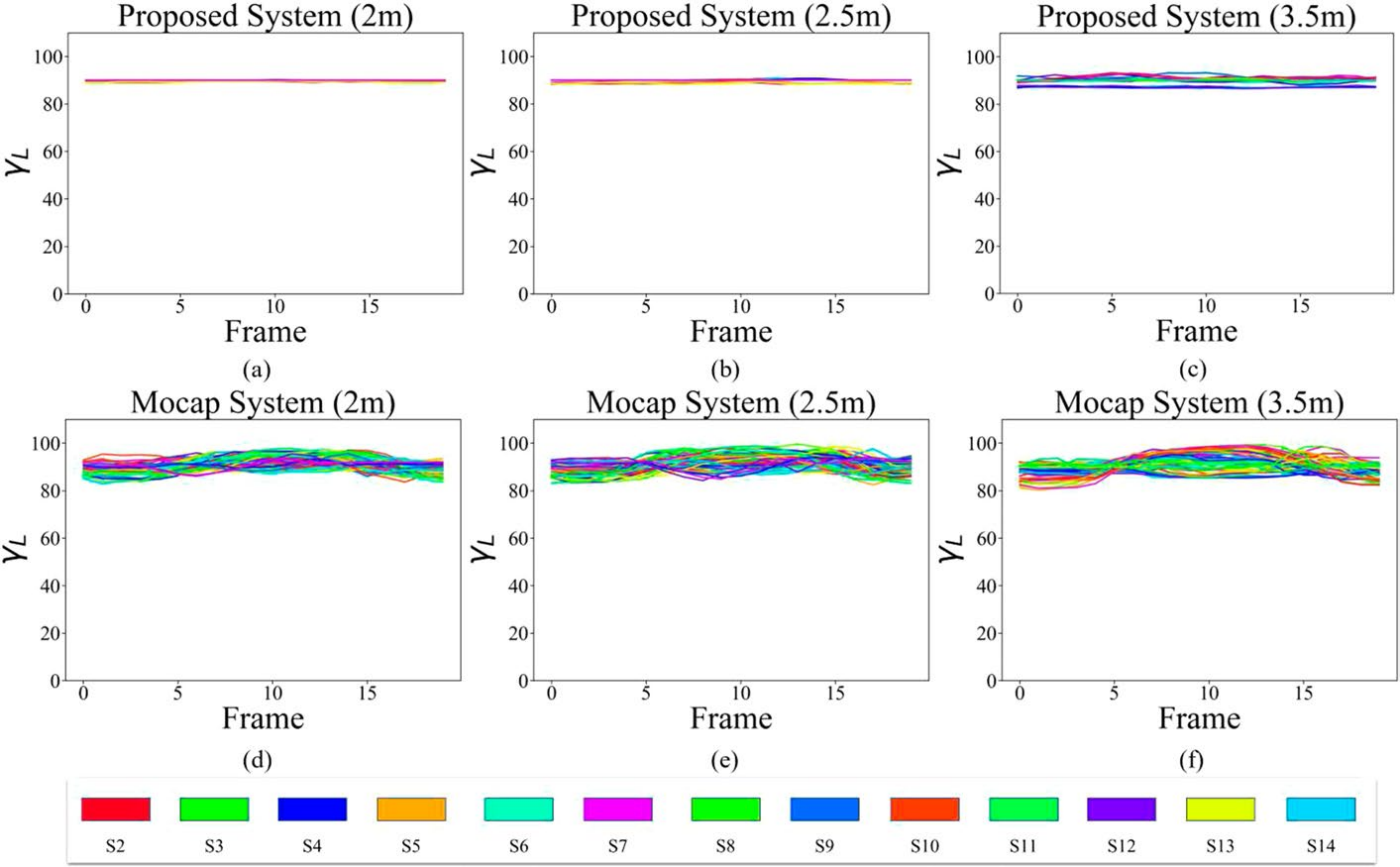
Signal $\gamma _{L}$ from proposed system at **a** 2 m, **b** 2.5 m, and **c** 3.5 m distances, and from Mocap at **d** 2 m, **e** 2.5 m, and **f** 3.5 m distances

Given that all subjects (S1–S14) were young and healthy, the changes in $\beta$ and the angle that the arm makes with the body in the transverse plane ($\gamma$) for SA exercise are expected to be zero. The difference in the measurement of these two angles from the two systems is mostly associated with the noise. As a result, the statistical tests are only applied and reported for $\alpha$ of both arms, which sweep from $0^\circ$ to $90^\circ$. The signals for angles $\alpha$, $\beta$, and $\gamma$ for all 13 participants standing at 3 different distances are plotted for the left arm in Figs. 4, 5, and 6, respectively. We use the L and R subscripts to refer to the $\alpha$, $\beta$, and $\gamma$ angles for the left and right arms, respectively.

Figure 4 demonstrates that the higher the distance of the subject from the camera is, the higher the error values are in the shoulder angle calculation. Furthermore, the standard deviation of the signals for different participants clearly increases as there is less homogeneity observed in Fig. 4b and c compared to a. Different participants also have the same pattern of signals. The only differences are in the slope of the signal, which indicates the difference in speed as well as in the magnitude of the abduction.

The participants’ elbows were extended during the exercise. Consequently, the ideal expected angle for $\beta$ is $180^\circ$. In the proposed system, the location of the elbow joint is not fixed over consecutive frames, as the SDK predicts the joints’ locations independently in each frame. This inconsistency resulted in a fluctuation in the elbow signal, as shown in Fig. 5a–c, where in some cases, the angle $\beta$ reaches less than $160^\circ$ (S6 in Fig. 5). For the Mocap system, the range of fluctuation is much lower, as shown in Fig. 5d–f. The initial offset of approximately $20^\circ$ in angle $\beta$ in Mocap is due to the physical location of the elbow marker, which was on the back of the elbow and is at a lower elevation compared to the shoulder markers (highlighted in Fig. 2).

As shown in Fig. 6a–c, the angle $\gamma$ for the proposed system is very close to the expected value of $90^\circ$, as the participants did not move their arm in the transverse plane toward or away from the camera. These figures also highlight the impact of the distance from the camera, as the higher the distance of the subject from the camera, the higher the variations are in shoulder angle in the z dimension. For the Mocap, however, we can see a higher deviation from 90^∘^, especially at the beginning and the end of the exercise, when the arm is dropped by the side of the body and is more likely not to be a perfect 90^∘^. It should be noted that we moved the subjects to different distances from the depth camera while the camera remained in a constant location. Therefore, the distance of the subject from the front Mocap cameras was also changed. This might be the reason for more fluctuations at higher distances for Mocap in Fig. 6d–f.

The obtained accuracy and deviations are influenced by several factors. Key among these are the limitations in accuracy and resolution of LIDAR and SDKs. Our system finds it challenging to capture fine joint movements with the same precision as the Mocap system. In addition, environmental factors such as changes in ambient light, reflective surfaces, or interference introduce noise to our system, impacting data quality and angle estimations.

In addition, differences in body structures and movement styles among participants could affect angle estimations. Despite our efforts to synchronize the two signals for an accurate comparison, timing differences between them may result in misalignments, which could impact the precision of our estimations.

**Fig. 7:**
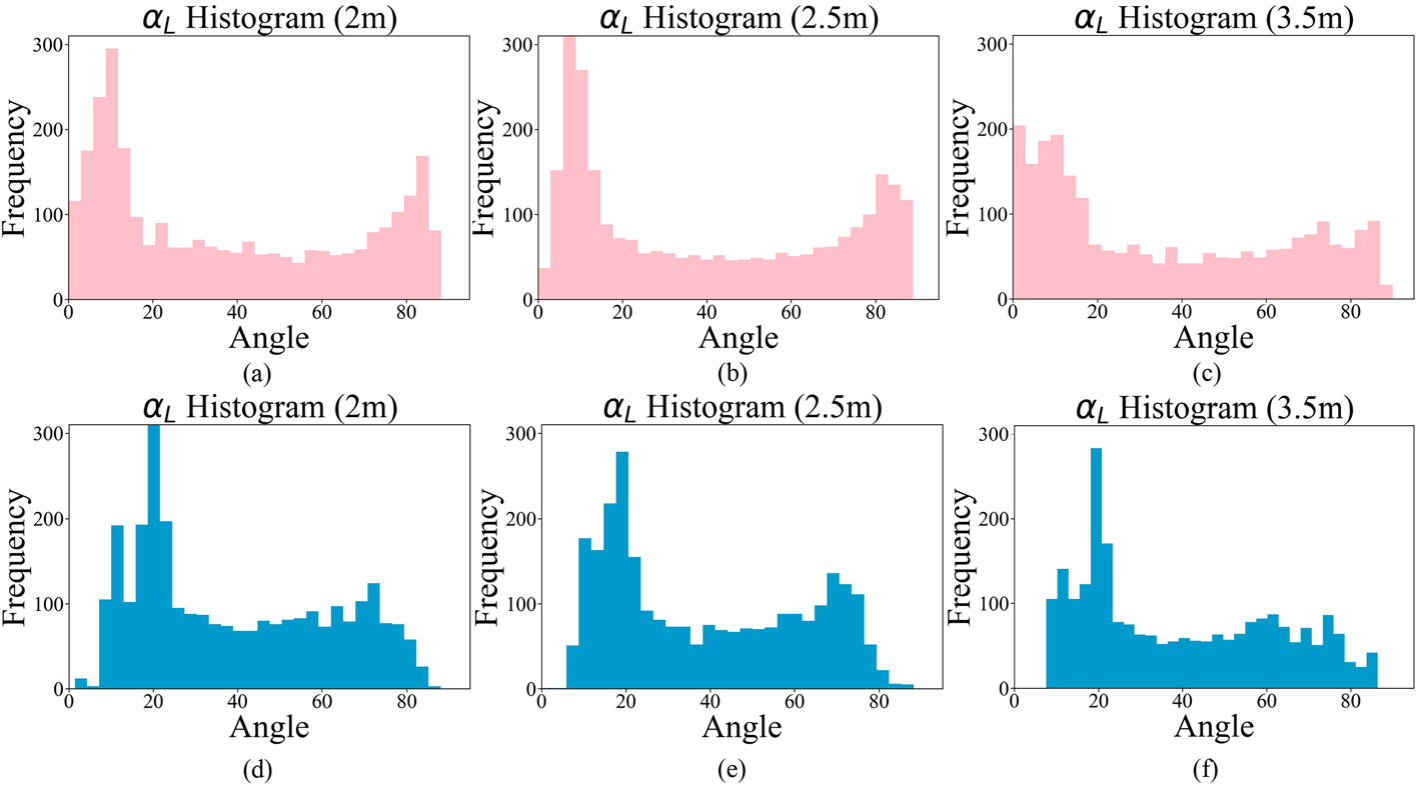
Histograms of $\alpha _{L}$ from the proposed system at distances of **a** 2 m, **b** 2.5 m, and **c** 3.5 m, and from Mocap at distances of **d** 2 m, **e** 2.5 m, and **f** 3.5 m

**Fig. 8:**
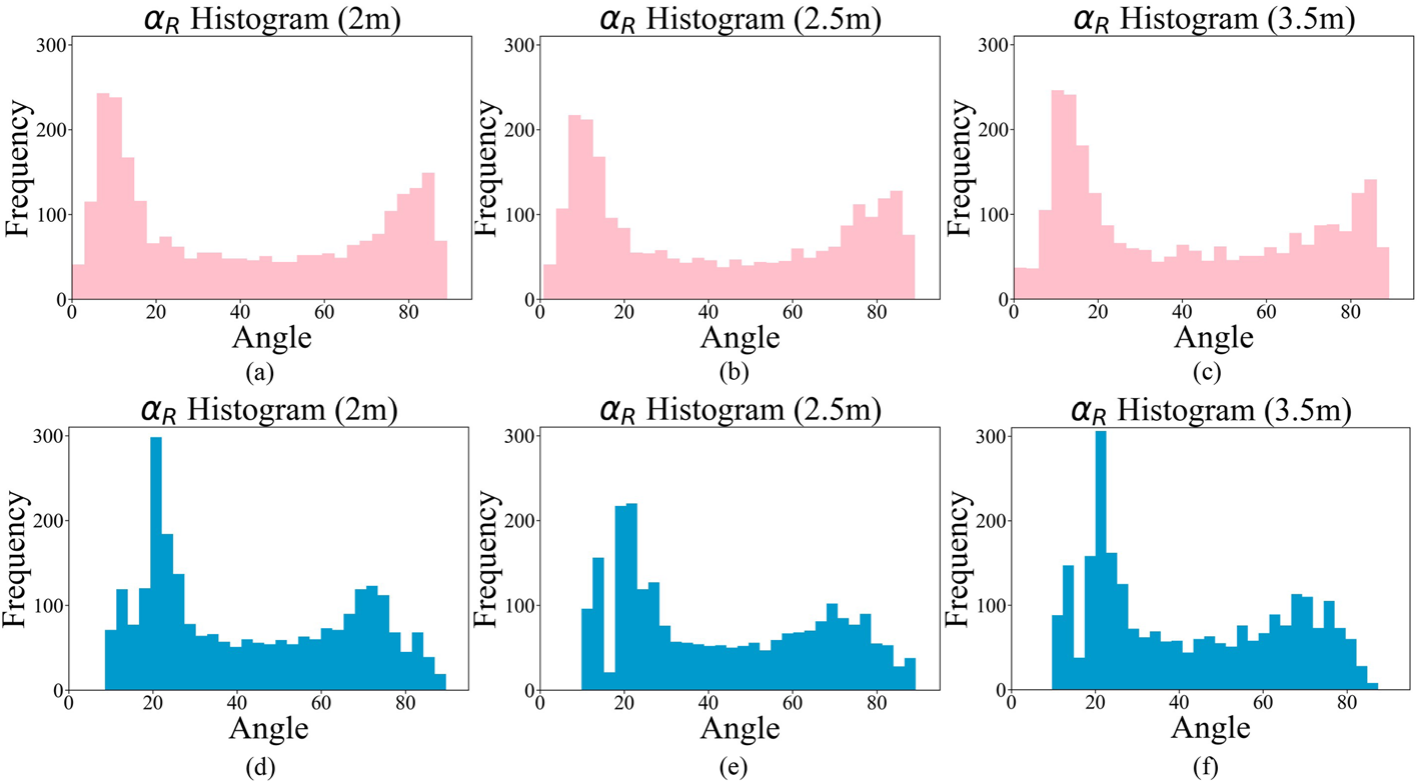
Histograms of $\alpha _{R}$ from the proposed system at distances of **a** 2 m, **b** 2.5 m, and **c** 3.5 m, and from Mocap at distances of **d** 2 m, **e** 2.5 m, and **f** 3.5 m

Furthermore, individual anatomical variations among participants, alongside their unique movement patterns, may influence observed deviations in angle estimations. In addition, timing disparities or latency in data capture and processing lead to misalignments, affecting the synchronization of estimations,

The angles $\beta$ and $\gamma$ have similar uniform distributions. The histograms of $\alpha _{L}$ during SAL and SAR exercises acquired by Mocap and Cubemos in all 3 distances and 13 subjects are shown in Figs. 7 and 8, respectively.

**Fig. 9:**
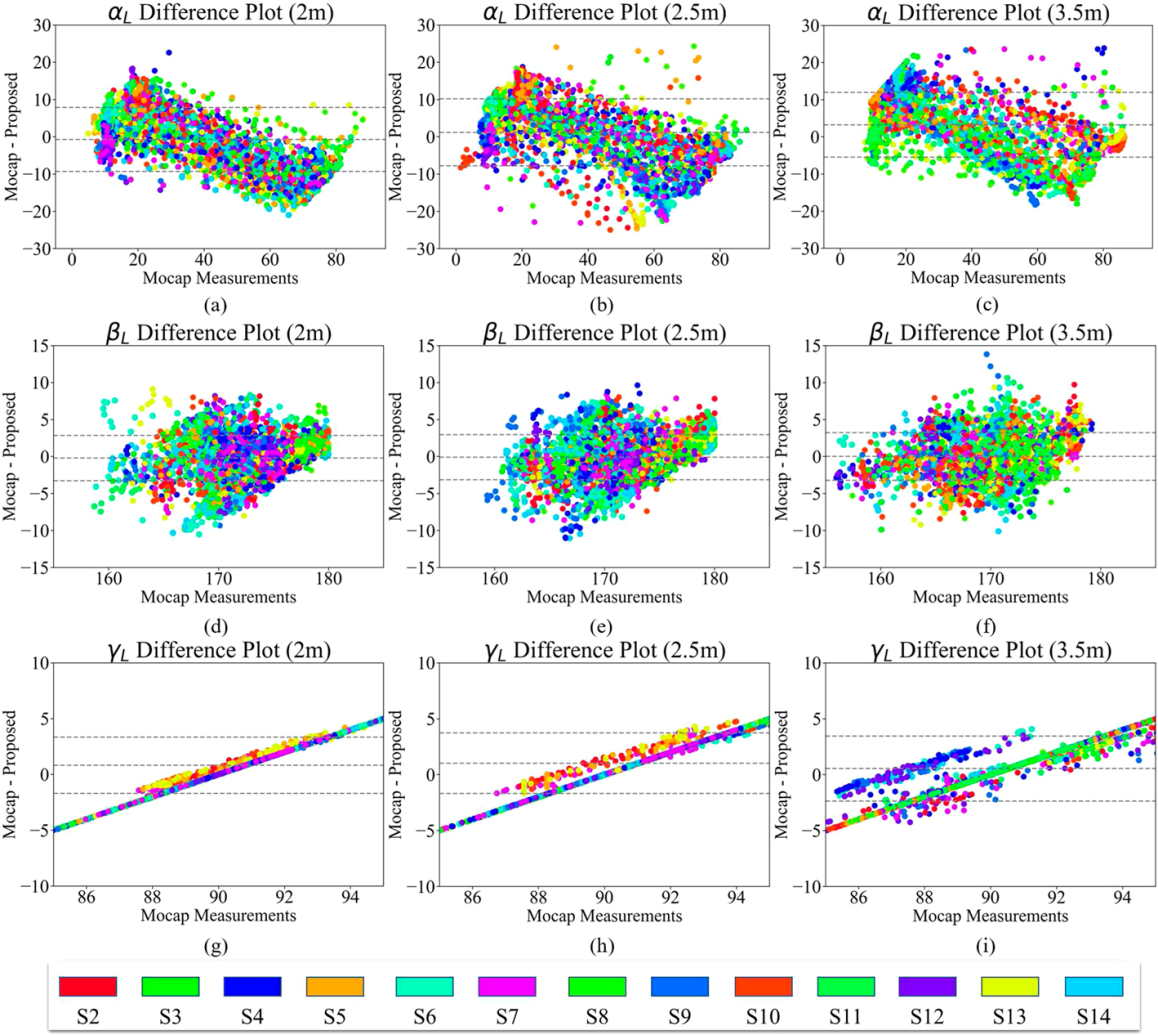
Difference plots for $\alpha _{L}$ at **a** 2 m, **b** 2.5 m, and **c** 3.5 m; for $\beta _{L}$ at **d** 2 m, **e** 2.5 m, and **f** 3.5 m; and for $\gamma _{L}$ at **g** 2 m, **h** 2.5 m, and **i** 3.5 m. The upper and lower dashed lines represent the standard deviation, and the middle dashed line shows the mean difference between the two systems

**Fig. 10:**
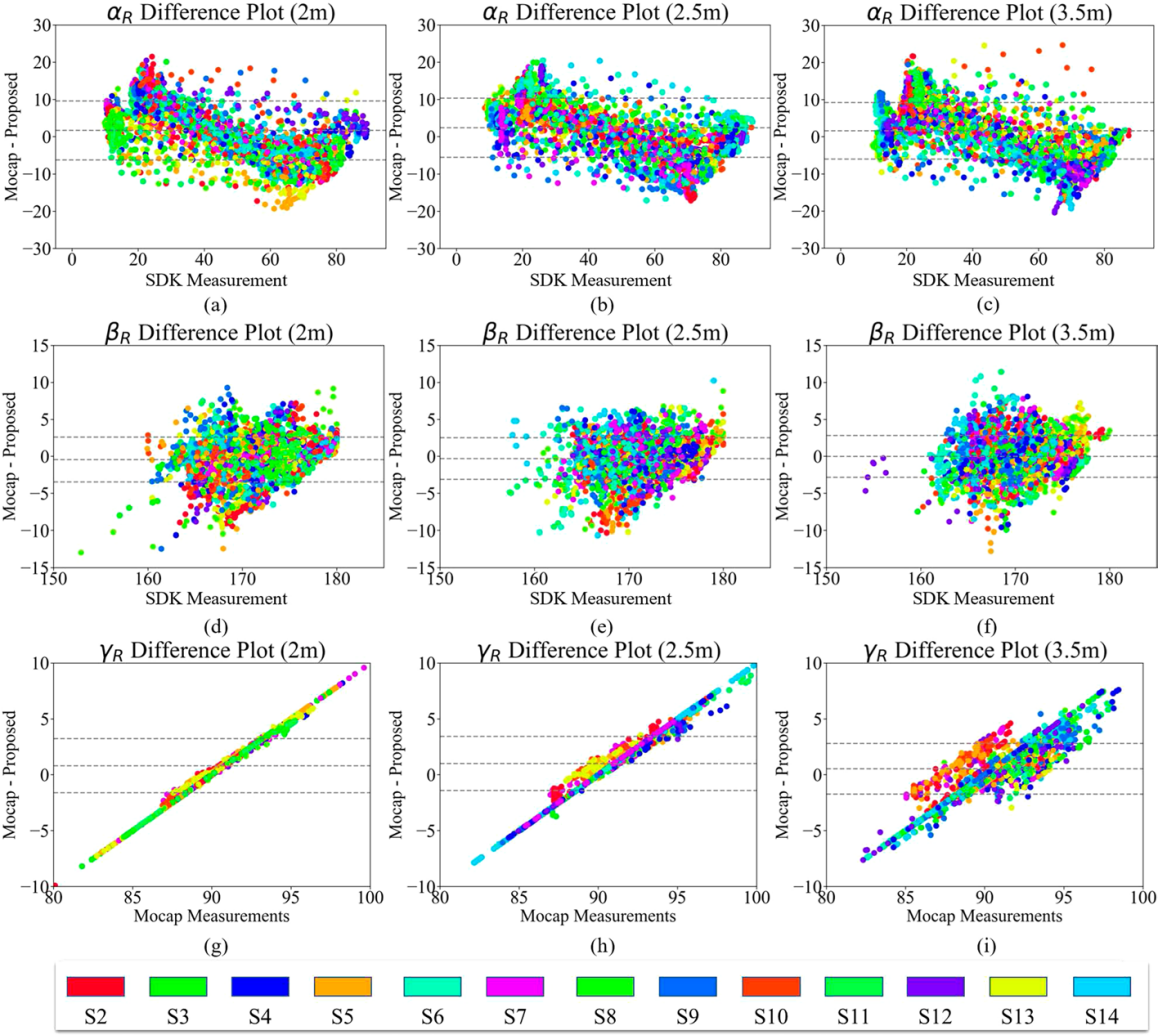
Difference plots for $\alpha _{R}$ at **a** 2 m, **b** 2.5 m, and **c** 3.5 m; for $\beta _{R}$ at **d** 2 m, **e** 2.5 m, and **f** 3.5 m; and for $\gamma _{R}$ at **g** 2 m, **h** 2.5 m, and **i** 3.5 m. The upper and lower dashed lines represent the standard deviation, and the middle dashed line shows the mean difference between the two systems

Given the non-normal distribution of the angles, a Friedman’s nonparametric test [32] was conducted on the angle data collected from the 3 distances. The test resulted in a *p* value less than 0.0001, indicating that at least two out of 3 distances have a statistically significant difference. The post-hoc analysis further demonstrated that the 2 m and 3.5 m distances from the camera resulted in a significant *p* value for all the angles. A Wilcoxon signed ranked test found a significant difference (*p* value < 0.001) between the right and left shoulder angles ($\alpha _{R}$ and $\alpha _{L}$) at all 3 distances. Other studies, such as [33], also found such a difference in the active range of shoulder flexion between the right and left shoulders. This difference might be because every participant had a dominant arm. In addition, Shapiro–Wilk and Kolmogorov–Smirnov tests were applied to check the normality of the errors between the Mocap and the proposed system. Both tests reject the null hypothesis, indicating that the errors are not normally distributed. Therefore, to compare the two measurements, we showed the difference plots in Figs. 9 and 10 for the left and right arms, respectively.

Figures 9 and 10 suggest that the proposed system could estimate all angles with low average error compared to the Mocap, whereas this error increases for the extreme cases. Looking at either small or large angles (e.g., $20^\circ$ and $70^\circ$ in $\alpha$), the proposed system provides either much higher or lower angles compared to the Mocap, respectively. This trend does not exist for $\beta$ (Figs. 9d–f and 10d–f) because, as mentioned, the signal-to-noise ratio (SNR) is low for the elbow angle fluctuations. In addition, the plots also showed the effect of distance from the depth camera. In other words, the standard deviation becomes more scattered and larger as the participant moves farther from the camera. The closest distance to the camera shows a denser plot compared to farther distances. This observation is consistent with the results of the statistical test applied.

The angle $\gamma$, shown in Figs. 9 and 10g–i, has a unique pattern. The Cubemos SDK is noticeably consistent in reporting $\gamma$. As a result, all the calculated angles have a value close to 90^∘^. Therefore, for different measurements of the Mocap, the proposed system reports the same value of approximately 90^∘^. The precision of the SDK is higher for the angle in depth. The reason for such a high precision is the laser technology used in the LiDAR ranging procedure. Figures 9 and 10 also showed that the difference plots of the right arm yielded a similar result aligned with the left arm angles. The previously mentioned trends and patterns related to the distance and magnitude of the angle are also visible in the right arm angles. There was no significant difference in the pattern of the angles calculated for the two arms.

The Mean Absolute Error (MAE), Root Mean Square Error (RMSE), coefficient of determination (R^2^), and Spearman correlation coefficient for all 5 repetitions across all 13 participants are reported in Tables 1 and 2 [34].

**Table 1:** MAE and RMSE results for the proposed system using Cubemos SDK

Angle	MAE (^∘^)			RMSE (^∘^)		
	2 m	2.5 m	3.5 m	2 m	2.5 m	3.5 m
$\alpha _{L}$	8.89 ± 1.09	10.08 ± 3.61	10.28 ± 4.40	10.30 ± 1.43	11.86 ± 4.21	12.33 ± 5.66
$\beta _{L}$	2.07 ± 0.78	2.20 ± 0.75	2.32 ± 0.61	2.53 ± 0.89	2.66 ± 0.86	2.85 ± 0.71
$\gamma _{L}$	2.04 ± 0.054	2.49 ± 0.54	1.90 ± 0.44	2.40 ± 0.55	2.85 ± 0.78	2.40 ± 0.55
$\alpha _{R}$	8.91 ± 3.53	9.88 ± 4.14	12.18 ± 8.14	10.44 ± 1.05	11.67 ± 4.65	14.72 ± 10.50
$\beta _{R}$	2.69 ± 0.96	2.23 ± 0.84	2.32 ± 0.63	3.16 ± 1.09	2.66 ± 0.92	2.91 ± 0.80
$\gamma _{R}$	1.57 ± 0.67	2.01 ± 0.65	1.37 ± 0.47	1.90 ± 0.75	2.39 ± 0.69	1.64 ± 0.55

**Table 2:** $\hbox {R}^{2}$ and Spearman correlation coefficient results for the proposed system using Cubemos SDK

Angle	$\hbox {R}^{2}$ (%)			Spearman coefficient (%)		
	2 m	2.5 m	3.5 m	2 m	2.5 m	3.5 m
$\alpha _{L}$	99.04	98.68	98.35	96.80	96.62	95.34
$\alpha _{R}$	98.88	98.78	98.53	96.81	96.52	96.24

The results indicate that there is a decreasing trend in the accuracy of SDK as the participant moves further from the LiDAR camera. The maximum error for the shoulder angle ($\alpha$) is always less than $10^\circ$ which is required by clinicians. Moreover, it suggests that the angle signal acquired from the SDK is highly similar to Mocap based on the correlation coefficients and $\hbox {R}^{2}$. For the $\hbox {R}^{2}$ and correlation coefficient, we only reported the values of the shoulder angle $\alpha$, since $\beta$ and $\gamma$ signals are not changing during the performance of the exercises and their fluctuations were only associated with the noise. Finally, the results showed that the right and left limbs had a similar outcome, as expected since these were healthy participants.

Mediapipe is an accepted conventional framework for human movement recognition and tracking and was studied as the second SDK in our experiment. We followed the same procedures for comparing Mediapipe with Mocap as we did for Cubemos. The MAE, RMSE, $\hbox {R}^{2}$, and correlation coefficients for Mediapipe are reported in Tables 3 and 4.

**Table 3:** MAE and RMSE results for the proposed system using Mediapipe framework

Angle	MAE (^∘^)			RMSE (^∘^)		
	2 m	2.5 m	3.5 m	2 m	2.5 m	3.5 m
$\alpha _{L}$	7.42 ± 1.18	8.79 ± 2.44	14.34 ± 9.18	8.56 ± 1.39	9.75 ± 2.40	16.19 ± 9.67
$\beta _{L}$	1.87 ± 0.65	1.74 ± 0.59	3.05 ± 2.58	2.22 ± 0.71	2.13 ± 0.67	3.05 ± 2.58
$\gamma _{L}$	1.64 ± 0.36	1.77 ± 0.62	2.22 ± 1.24	2.08 ± 0.42	2.26 ± 0.71	2.66 ± 1.45
$\alpha _{R}$	8.48 ± 2.67	9.62 ± 3.14	15.63 ± 11.34	9.87 ± 3.09	11.12 ± 3.51	17.32 ± 12.16
$\beta _{R}$	2.35 ± 1.30	2.20 ± 1.27	8.95 ± 7.15	2.78 ± 1.58	2.68 ± 1.71	11.14 ± 8.90
$\gamma _{R}$	1.97 ± 0.89	1.85 ± 0.88	5.53 ± 4.65	2.33 ± 0.96	2.28 ± 1.09	6.74 ± 5.41

**Table 4:** $\hbox {R}^{2}$ and and Spearman correlation coefficient results for the proposed system using Mediapipe framework

Angle	$\hbox {R}^{2}$ (%)			Spearman coefficient (%)		
	2 m	2.5 m	3.5 m	2 m	2.5 m	3.5 m
$\alpha _{L}$	98.90	98.27	94.80	97.52	96.47	96.10
$\alpha _{R}$	98.67	98.68	93.06	97.20	96.25	94.17

For Mediapipe, the overall trend was the same as that for Cubemos. However, there were some slight differences. First, the Mediapipe had a generally poorer performance compared to Cubemos. The reason could be that Cubemos was designed to work with an Intel RealSense device, which we are using in our study. Second, the difference between 2 m and 2.5 m from the camera is much less evident in Mediapipe. It still shows a noticeable difference at 3.5 m, but for the 0.5 m difference in distance, the results are quite similar. Last, the effect of distance on precision was greater in Mediapipe.

The precision of our system drops as the distance from the LiDAR camera increases, which has practical implications. This reduced precision can make joint angle estimations less reliable, particularly when participants are far from the LiDAR camera. Due to technological constraints, it might not be feasible to completely resolve this loss of precision at longer distances.

Thus, defining an acceptable operating range becomes essential for the users. Integrating this information into system interfaces and user manuals, and providing alerts for users operating beyond the recommended range can enhance awareness and adherence to optimal conditions.

To enhance the precision of joint angle estimations at extended distances, optimization strategies could involve exploring alternative sensors with higher resolutions or employing advanced signal processing techniques to compensate for reduced data quality at longer distances.

To enhance the precision of joint angle estimations at extended distances, optimization strategies could involve exploring alternative sensors with higher resolutions or employing advanced signal processing techniques to compensate for reduced data quality at longer ranges.

### Calibration results

**Fig. 11:**
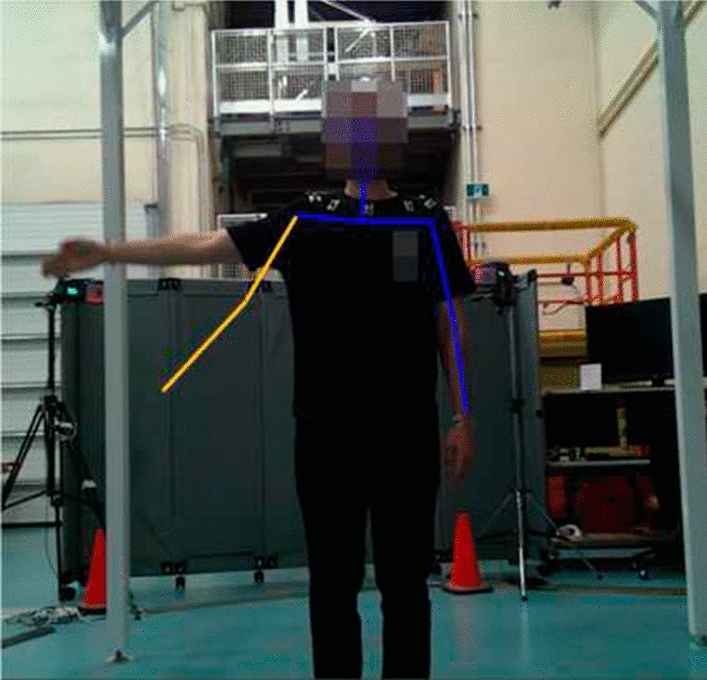
A sample frame in which the Cubemos SDK failed to detect the elbow and wrist joints due to fast movement

**Fig. 12:**
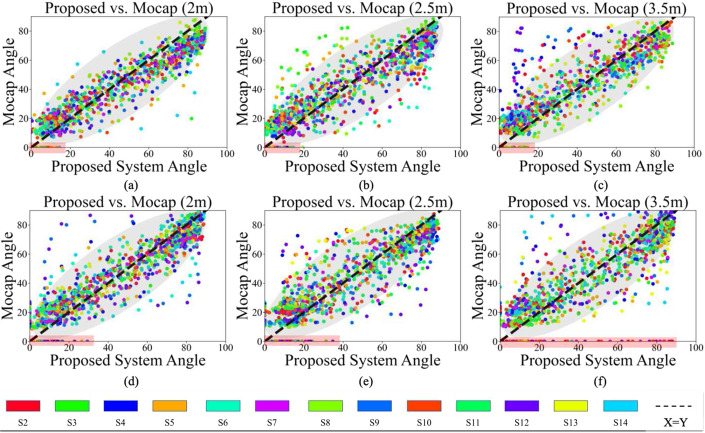
Proposed system vs. Mocap plots for $\alpha _{L}$ at **a** 2 m, **b** 2.5 m, and **c** 3.5 m and for $\alpha _{R}$ at **d** 2 m, **e** 2.5 m, and **f** 3.5 m

One of our observations when using the proposed system was that when subjects performed exercises at a fast pace, the motion area within the frames exhibited blurriness, resulting in challenges for the SDK to accurately detect joint positions. In these cases, as mentioned in “Method” section, we kept the previous locations of the joints. An example is depicted in Fig. 11. These instances are visually highlighted in Fig. 12 with a red area.

This observation becomes evident in cases where the Mocap system indicated a joint angle of $0^\circ$, whereas our proposed system recorded angles ranging between $0^\circ$ and $20^\circ$ for the left arm and $0^\circ$ and $90^\circ$ for the right arm. This loss of information within the proposed system is irreversible and cannot be fixed with calibration methods. However, other data points highlighted with the gray area in Fig. 12 show a pattern closely resembling the ideal system. Therefore, a regression analysis is employed to calibrate the system and minimize the associated errors.

Figure 12 shows the dot plots of the shoulder angle (in the range of [$0^\circ$, $90^\circ$]) captured from the proposed system versus Mocap. This figure provides information on how the two angle signals change with respect to each other. These figures show that the closer the camera was to the subject, the more linear the trend (Fig. 12a). In other words, the angles $\alpha$ from Mocap and the proposed system seem to have a linear relationship, which will help further calibrate the system.

#### Cubemos SDK-based system calibration results

**Fig. 13:**
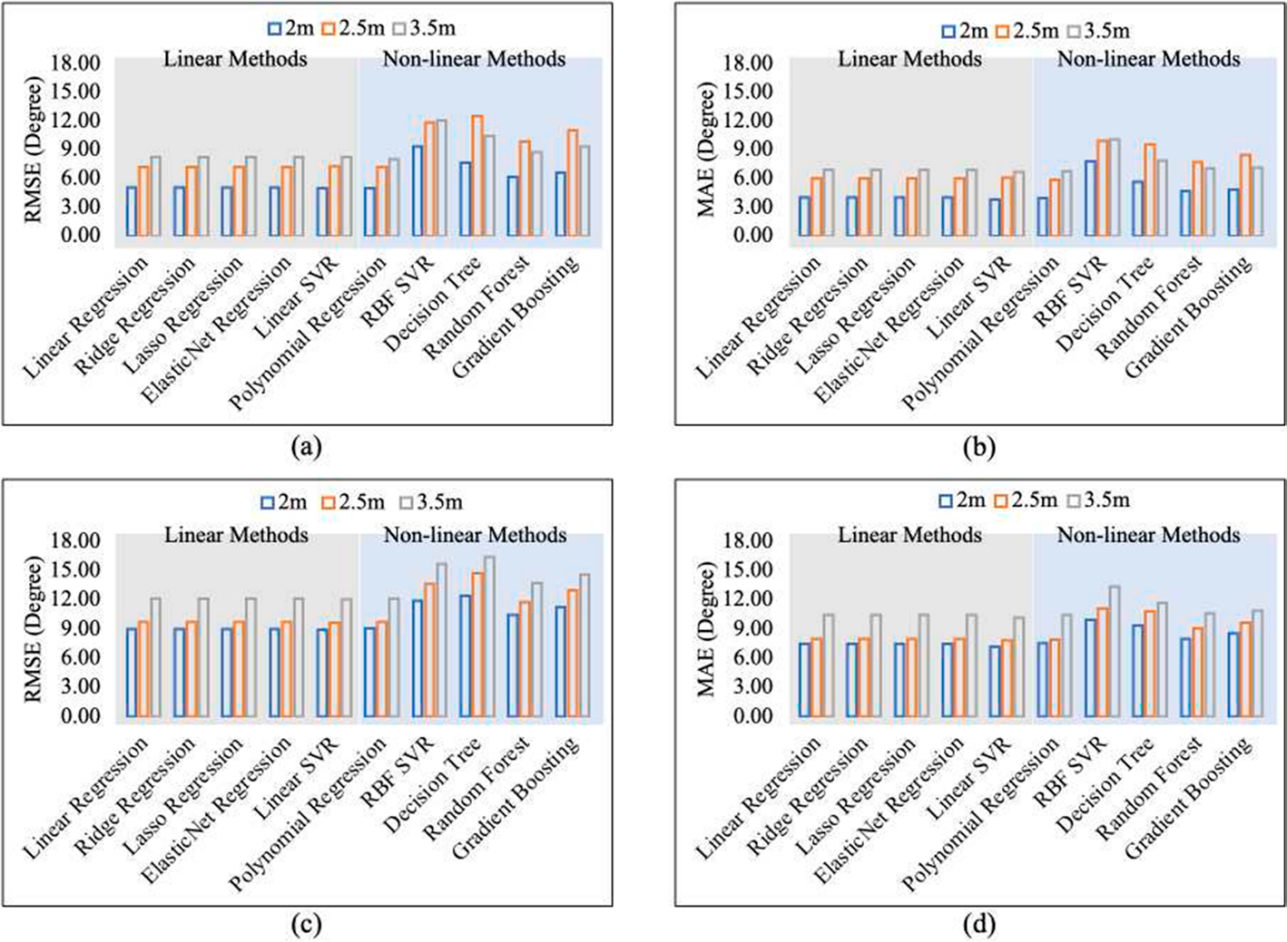
Regression results as **a** RMSE and **b** MAE for $\alpha _{L}$ and **c** RMSE and **d** MAE for $\alpha _{R}$

The Cubemos results that showed a better performance and lower errors were chosen for the calibration. Figure 13 demonstrates the regression results using the 10 regression models for both SAL (Fig. 13a and b) and SAR (Fig. 13c and d).

When comparing the SAL results shown in Fig. 13a and b with SAR in Fig. 13c and d, we see that the errors decrease more for the left arm ($\alpha _{L}$) than for the right arm ($\alpha _{R}$) after calibration. This could be because there was a stronger linear relationship between the Mocap data and our proposed system, as shown in Fig. 12.

Overall, the linear models shown in the gray-shaded area in Fig. 13 provided lower overall RMSE and MAE compared to the nonlinear models shown in the blue-shaded area in Fig. 13. Linear Regression (LR), Ridge Regression (RR), Lasso Regression (LaR), and Elastic net Regression (ER) models exhibit identical performance in both RMSE and MAE. The coefficients of the polynomial regression terms with powers are notably small, resulting in a model that closely resembles linear regression. This suggests that the regularization techniques used in LaR, RR, LR, and ER did not significantly affect their performance compared to the simpler linear regression model.

For all models, a larger distance from the camera corresponded to higher RMSEs and MAEs for the right hand. This indicates that the observed trend persists even after the calibration process. Nevertheless, this pattern was not the same for the left hand. In the case of the Decision Tree (DT), Random Forest (RF), and Gradient Boosting (GB) models, the 3.5 m distance resulted in lower RMSE and MAE compared to the 2.5 m distance. However, the lowest errors were consistently associated with the closest distance of 2 m from the camera. The linear Support Vector Regression (SVR) worked best for calibration at 2 m, whereas the linear models performed equally well for 2.5 m and 3.5 m distances from the camera. This shows that we should adjust our calibration method based on how far the patient is from the camera. We can obtain this information simply from our camera’s third dimension (depth-z).

The initial difference between the MAE and RMSE values before calibration indicates a potential bias in our predictions. Since MAE is less affected by outliers than RMSE, a higher MAE indicates the larger errors on average. After applying calibration, we effectively addressed this bias, leading to improved overall model performance.

**Fig. 14:**
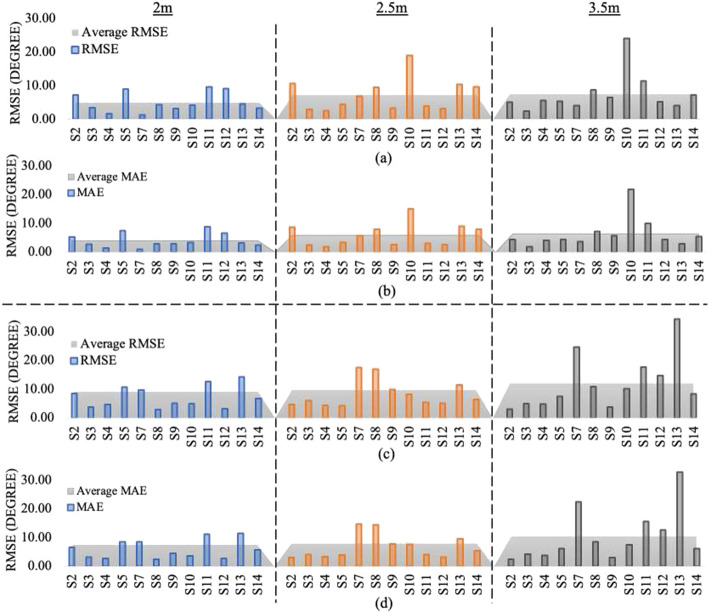
MAE and RMSE at all 3 distances for **a** $\alpha _{L}$ before calibration, **b** $\alpha _{L}$ after calibration, **c** $\alpha _{R}$ before calibration, and **d** $\alpha _{R}$ after calibration

Figure 14 demonstrates the subject-by-subject results before (Fig. 14a and c) and after calibrations (Fig. 14b and d). These figures highlight the proposed technique’s effectiveness in accommodating diverse participants with different physical characteristics, exercise patterns, ranges of motion, and initial errors.

**Fig. 15:**
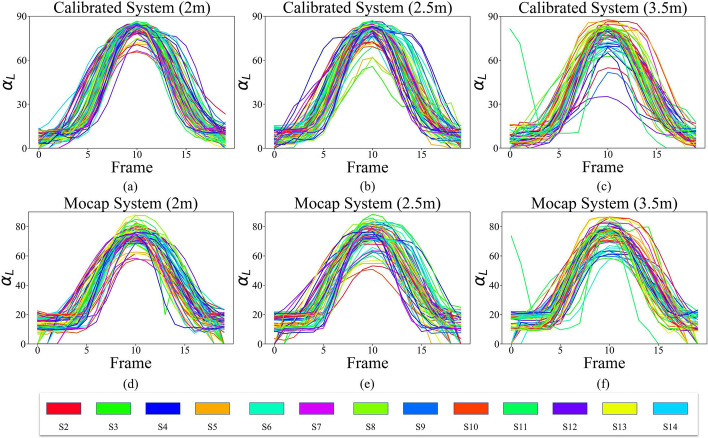
Signal $\alpha _{L}$ from the calibrated system at **a** 2 m, **b** 2.5 m, and **c** 3.5 m distances, and from Mocap at **d** 2 m, **e** 2.5 m, and **f** 3.5 m distances

Figure 15a–c illustrates the ($\alpha _{L}$) values obtained from the calibrated system in direct comparison with the corresponding data acquired from the mocap (d–f).

## Conclusion and future work

This experiment showed the potential of a marker-less Mocap system in tracking body joints and calculating different upper-limb angles. The signals acquired from the proposed and ground truth systems were highly correlated and followed the same patterns. All MAEs were less than $10^\circ$. The depth feature of the camera is accurate and helpful in calculating in-depth angles that cannot be achieved using a regular RGB camera. The findings also suggest a negative correlation between the distance from the camera and the accuracy of the system. In other words, the farther the participant stands from the camera, the higher the angle estimation error will be.

The results of our experiment showed a better overall performance for the Cubemos SDK compared to the Mediapipe. We hypothesized that this is because the Cubemos SDK was created to work with the hardware we have used in this study, whereas Mediapipe is a more general framework.

For the calibration part, the evaluation of the 10 regression models revealed that the polynomial regression model achieved the best results, while the DT model performed the poorest. In addition, the LR, RR, and LaR models demonstrated similar performance.

Although the outcomes were highly positive, our study does have certain limitations. The SDK-based system will not behave as expected for exercises that require the participants to point toward the camera. The available SDKs are trained in such a way that they recognize the human body facing the camera, but as soon as a joint becomes hidden from the camera, the data will be either lost or highly inaccurate.

We further recognized that the positioning of the camera is crucial, especially for exercises that need to be performed in laying down positions. Another limitation that we observed in this experiment is that the Mocap markers move during the trials. Therefore, we will have erroneous angle creation, which will cause a lower accuracy in the final comparison of the systems.

The placement of Mocap markers had a direct impact on the results. We took care to be as consistent as possible with locating markers. For future studies, we recommend identifying the region of interest of each joint from the SDK so that the Mocap markers are attached to those specific locations for a fair comparison.

Regarding the future work and the potential implications of our study for the field of tele-rehab, our study points toward a potential avenue for automating range of motion assessments in clinical settings, signaling a prospective advancement in tele-rehab technology. Future research directions should delve deeper into refining calibration methods and enhancing hardware–software integration to address limitations and further improve accuracy. Moreover, the identified correlation between participant distance and system accuracy requires future research to explore strategies for mitigating this challenge.

## Methods

### System setup and data collection

**Fig. 16:**
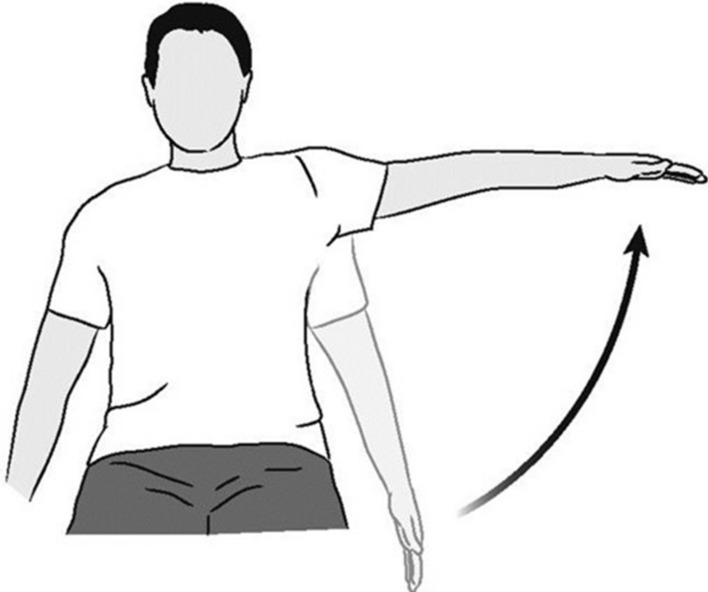
A figure burrowed from [35], showing shoulder abduction exercise

We conducted our data collection at the KITE Research Institute—Toronto Rehabilitation Institute (TRI), University Health Network after obtaining approval from the Research Ethical Board (REB). Fourteen able-bodied participants consented to be recorded while performing 5 repetitions of shoulder abduction left and right (SAL & SAR) exercises, as shown in Fig. 16.

The demographic information of all 14 participants (8 females and 6 males) is shown in Table 5.

**Table 5:** Participant demographics

Subject #	Sex	Age	Height (m)	BMI (kg/m^2^)
S1	Female	22	1.59	23.73
S2	Female	25	1.68	21.26
S3	Male	24	1.75	29.39
S4	Female	22	1.62	20.19
S5	Female	26	1.68	19.49
S6	Male	28	1.80	24.69
S7	Male	28	1.92	24.41
S8	Male	27	1.75	27.75
S9	Female	23	1.65	22.04
S10	Male	22	1.75	21.22
S11	Female	22	1.75	26.12
S12	Male	28	1.70	25.95
S13	Female	23	1.60	21.48
S14	Female	26	1.65	22.04
Average	–	24.71 ± 2.34	1.71 ± 0.08	23.55 ± 2.85

The participants performed 3 sets of 5 repetitions while standing at 3 different distances of 2 m, 2.5 m, and 3.5 m from the LiDAR camera placed on a table 1 m above the ground. The Intel RealSense L515 [23] LiDAR camera was used for the data collection. The L515 includes Time of Flight (ToF) technology, offering high-resolution depth and 2D image capture with a range of up to 9 ms. It features a depth stream resolution of 1024 x 768 pixels and a 2D image resolution of 1920 x 1080 pixels, functioning at a maximum frame rate of 30 fps. With a field of view (FoV) of $70.4^\circ$ x $55^\circ$ (± $3^\circ$) and an accuracy of ±2% the L515 provided precise depth data for our experiment [23].

**Fig. 17:**
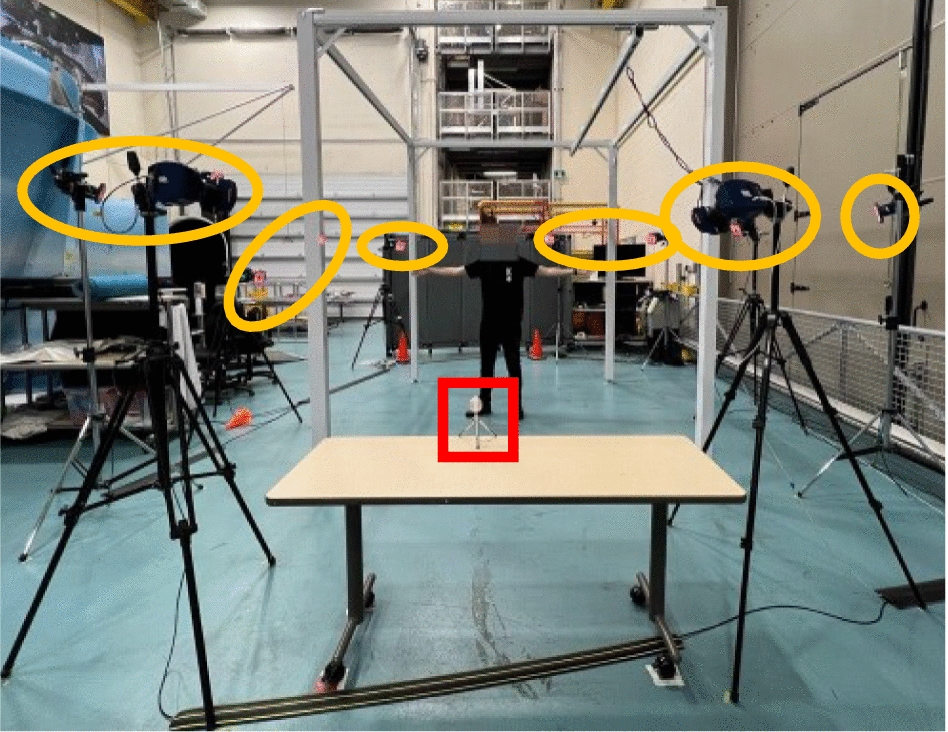
The KITE-TRI basement setup: participant standing, LiDAR camera (red square), and Mocap cameras (yellow ovals)

A $\text {Cortex}^\text {TM}$ motion analysis [28] with 10 time-of-flight sensors was used as the Mocap system for this experiment. These sensors, using ToF technology similar to the L515, track movements with high precision and accuracy. The frame rate we selected for our work was 256 fps. The setup is shown in Fig. 17.

**Fig. 18:**
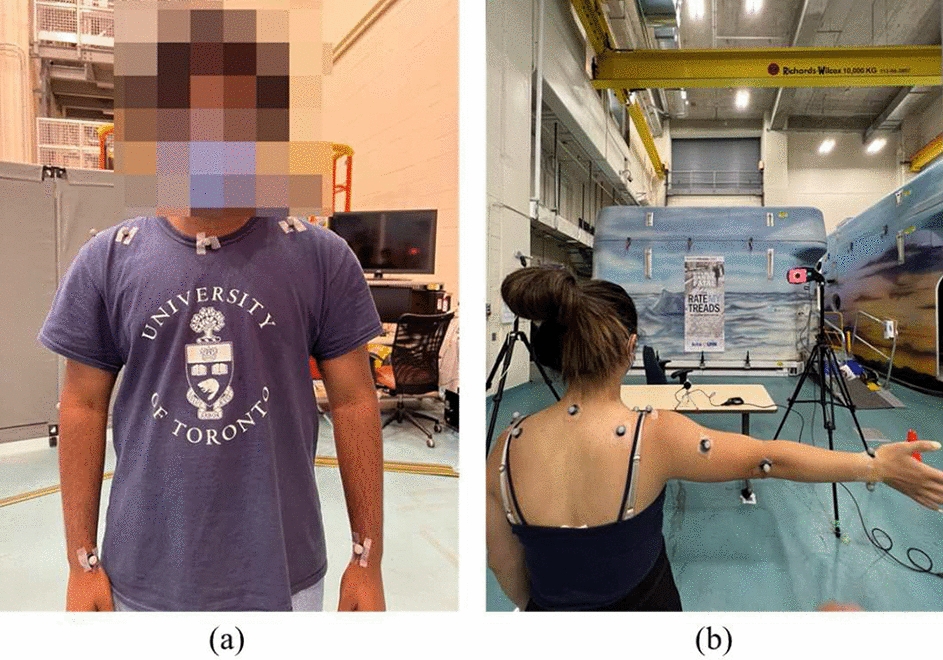
Photos from **a** front and **b** back of the participants with Mocap markers on

After consulting with a physiatrist, 17 Mocap markers were placed on participants’ upper extremities, including (i, ii) left and right ears, (iii) middle of the chest above sternum, (iv) on the back on C7, (v–x) for each left and right shoulder on top of the clavicle (collarbone), 10 cm from the top of the collarbone to the center of the chest on the collarbone, and 10 cm from the acromion process toward the middle of the spine, (xi, xii) on the back of the left and right elbow, (xiii–xvi) on the lateral sides of each left and right wrist near the radius and ulna connection to carpal bones, and (xvii) on the right arm to distinguish the right arm from the left arm later in the recordings. Figure 18 shows participants with Mocap markers attached to both the front and back of the body.

### Data analysis methodology

**Fig. 19:**
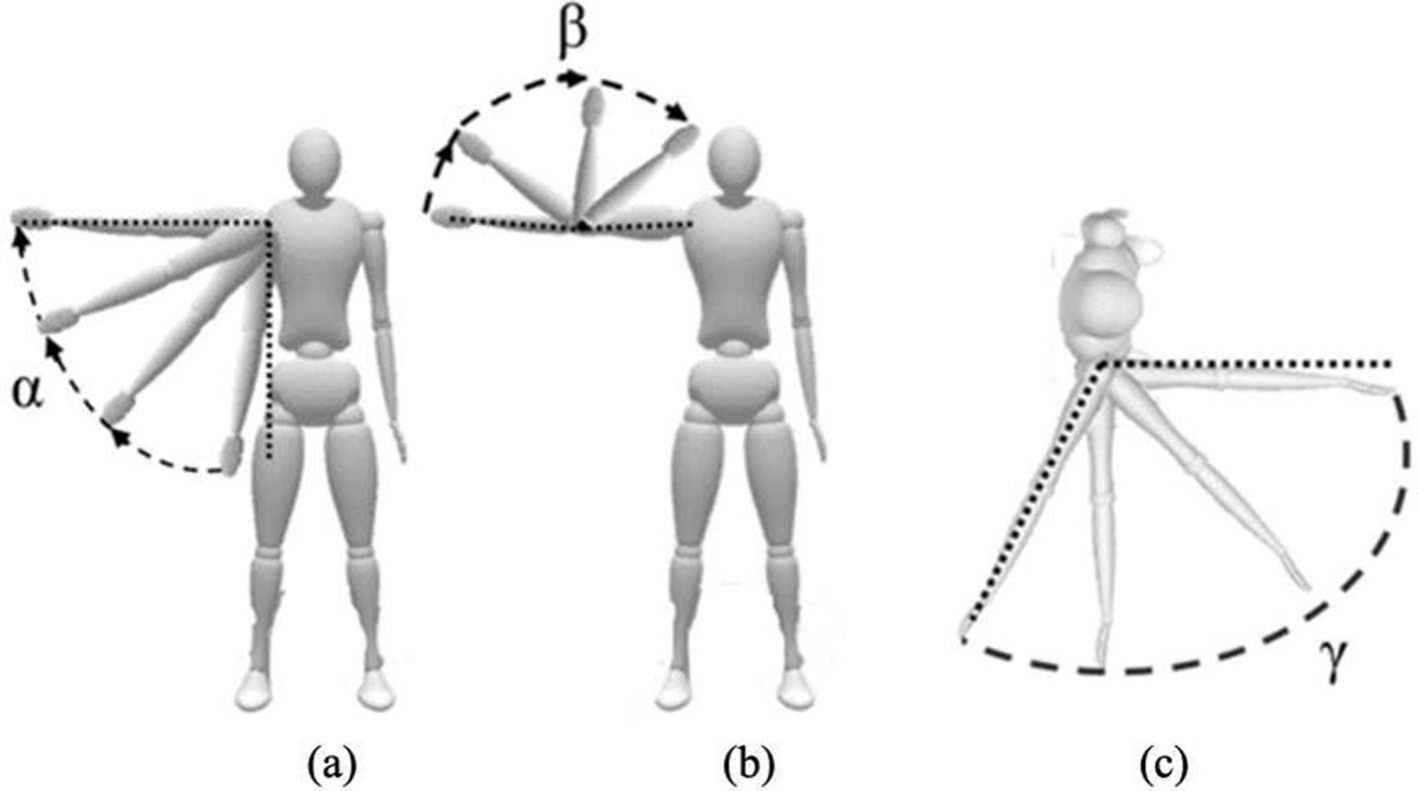
**a** Shoulder $\alpha$, **b** elbow $\beta$, and **c** arm–body $\gamma$ angles in the transverse plane

Given that the performed exercise is shoulder abduction, the angles $\alpha$ and $\beta$ were calculated from both Mocap and the LiDAR camera. Moreover, $\gamma$ was calculated with respect to the normal vector perpendicular to the participant’s frontal plane to validate the reliability of the system along the z-axis, which was acquired by the LiDAR camera. The angles are shown in Fig. 19.

Knowing that a correct repetition of SAL/SAR involves having the elbow and arm in the same plane as the body, the expected value for $\alpha$ should be from $0^\circ$ to a maximum of $90^\circ$, while $\beta$ should remain at approximately $180^\circ$, and $\gamma$ should be held at approximately $90^\circ$.

Plots that show the difference between the two methods on the vertical axis against the best estimate of the true value on the horizontal axis are used to compare our results with those of Mocap [36]. In addition, the correlation coefficient was obtained to determine whether both systems provide similar trends over time. Finally, a statistical test was conducted to determine whether there were any significant differences in the angle measurements when the distance from the camera changed.

#### Handling frames with missing joints data

In certain frames during the tracking process, the SDK may encounter difficulty in detecting specific joints of the participant’s arm. To address this situation, a strategy is employed wherein the joint data from the last successful detection are used for the problematic frame. In other words, the system will keep using the latest joint information acquired until the SDK successfully identifies the updated location of the joint. Upon detection, the newly obtained joint data are incorporated into the calculations, and simultaneously, the most recent joint data received are updated to correspond with the newly acquired data. This iterative procedure ensures continuity in joint tracking by seamlessly incorporating the most accurate and up-to-date information provided by the SDK.

### Calibration

In this paper, we present a subject-based calibration phase for the system that used the best-performing SDK. The aim is to minimize the system’s error in the calculation of the $\alpha$ angle for left and right shoulders. In this phase, we evaluate the performance of 10 different linear and nonlinear regression models, including LR, RR, LaR, and ER, linear SVR, as well as DT, Random Forest RF, GB, Polynomial Regression (PR) and SVR with the Radial Basis Function (RBF) kernel. We filtered the data based on the current subject, exercise, and distance and randomly split it into training and testing sets after shuffling. Eighty percent of the data were used for training, whereas 20% remained unseen to the model as the test set.

The key idea is that in tele-rehab, the system can be calibrated individually for each patient. This adjustment can take place during the initial visit with the doctor, where the patient receives their prescription. This visit can be a great opportunity to fine-tune the system according to the patient’s unique exercises and movement patterns. We evaluated our personalized calibration method using RMSE and MAE on the angle data.

Our study uses a personalized calibration method, where unique regressors are trained on each participant’s data. This approach ensures a custom-fit regressor for each individual, accommodating the varying degrees of musculoskeletal disorders present among patients. As a result, every participant benefits from a regressor model tailored to their specific data, enhancing both the accuracy and precision of the calibration process for individualized performance.

## Data Availability

Not applicable.
